# First Nations Australians’ experiences of current alcohol policy in Central Australia: evidence of self-determination?

**DOI:** 10.1186/s12939-022-01719-z

**Published:** 2022-09-08

**Authors:** Annalee E. Stearne, KS Kylie Lee, Steve Allsop, Anthony Shakeshaft, Michael Wright

**Affiliations:** 1 National Drug Research Institute and enAble Institute, Faculty of Health Sciences, Curtin University, WA Perth, Australia; 2 Faculty of Medicine and Health, Central Clinical School, The University of Sydney, NHMRC Centre of Research Excellence in Indigenous Health and Alcohol, Sydney, NSW Australia; 3 University of New South Wales, National Drug and Alcohol Research Centre, Sydney, NSW Australia; 4 Faculty of Health Sciences, School of Allied Health, Curtin University, WA 6845 Perth, Australia

**Keywords:** Alcohol, Self-determination, First Nations Australians, Policy, Northern Territory

## Abstract

Alcohol is the leading cause of healthy years lost. There is significant variation in alcohol consumption patterns and harms in Australia, with those residing in the Northern Territory (NT), particularly First Nations Australians, experiencing higher alcohol-attributable harms than other Australians. Community leadership in the planning and implementation of health, including alcohol, policy is important to health outcomes for First Nations Australians. Self-determination, a cornerstone of the structural and social determinants of health, is necessary in the development of alcohol-related policy. However, there is a paucity of published literature regarding Indigenous Peoples self-determination in alcohol policy development. This study aims to identify the extent to which First Nations Australians experience self-determination in relation to current alcohol policy in Alice Springs/Mbantua (Northern Territory, Australia).

Semi-structured qualitative yarns with First Nations Australian community members (*n* = 21) were undertaken. A framework of elements needed for self-determination in health and alcohol policy were applied to interview transcripts to assess the degree of self-determination in current alcohol policy in Alice Springs/Mbantua. Of the 36 elements, 33% were not mentioned in the interviews at all, 20% were mentioned as being present, and 75% were absent. This analysis identified issues of policy implementation, need for First Nations Australian leadership, and representation.

Alcohol policy for First Nations Australians in the NT is nuanced and complicated. A conscious approach is needed to recognise and implement the right to self-determination, which must be led and defined by First Nations Australians.

First Nations Australians’ experiences of current alcohol policy in Central Australia: evidence of self-determination?

## Background

Worldwide alcohol is the leading cause of healthy years lost in people aged 15–49 years [1]. Alcohol was estimated to cost Australians at least $33 billion AUD in 2017–18 (and as high as AUD $214 billion) [2]. In the Northern Territory (NT) Australia (2015–16), it was estimated that the social cost of alcohol was $7,577.94 per adult [3]. Alcohol consumption patterns also vary greatly across populations and jurisdictions [4]. One in 12 residents (Territorians) drink every day, compared to one in 20 in the wider Australian population [5]. Furthermore, First Nations Australians^1^ who drink, do so more often than their non-Indigenous counterparts (11 + drinks per occasion:11% versus 7%) [5, 6]. These are at levels that exceed both the single occasion and lifetime risk according to the Australian Guidelines [7].

As a result, the NT population and in particular First Nations Territorians, experience higher alcohol-attributable morbidity and mortality than other Australians [3, 8]. Alcohol-related deaths among First Nations Australians in Central Australia are more than three times the national rate (14 compared to 4.17 per 10,000; data only available from 2007) [9]. Further it this, between July 2015 and June 2017 alcohol-related hospitalisations in the NT (19.2/1000) are significantly higher than the national rate (9.1/1000) [10]. While there may be many factors that that influence this difference, including how hospitals define and record alcohol-related hospitalisations, it is not possible to identify the degree to which these factors affect the data. Nevertheless, NT residents experience alcohol-related harms at significant rates. These disparities, need to be understood within the wider context of First Nations Australians’ experiences of intergenerational trauma, colonisation, dispossession, and exclusion [11, 12].

First Nations Australians were prohibited from purchasing and consuming alcohol in the NT until the *Licensing Ordinance 1964* (NT) [13]. Whilst ending such discrimination is necessary, the sudden change increased the prevalence of alcohol use and related harms. A suite of harm minimisation strategies have been developed by and with First Nations Australians to reduce harms from alcohol [14]. For example, supply reduction strategies in Alice Springs/Mbantua (the largest town in Central Australia, NT) have included: the purchase and operation of a local drinking club by Tangentyere Council; the purchase of a liquor outlet by the Central Australian Aboriginal Congress and then their intentional cancelation of the liquor licence [15]; and broad community-level restrictions on the take-away sale of alcohol [16]. These strategies have been coupled with innovative community initiatives such as night patrols and sobering-up shelters [17].

Ensuring community involvement in the planning and implementation of health policy, including in relation to alcohol, is vital to positive health outcomes for First Nations Australians [18, 19, 20]. Self-determination, an internationally recognised right for Indigenous Peoples^2^, is a cornerstone of the structural and social determinants of health. Self-determination is challenging to define and means different things to different people in varying contexts [11, 21, 22, 23]. In this paper, we define self-determination as: “… the internationally recognised and on-going right of Indigenous Peoples to collectively determine their own pathway, within and outside of existing settler societies [20].” Edwards (1980) observed that prevention cannot be imposed on a society or a community – there needs to be an invitation to change – this is still relevant today particularly for marginalised groups [24]. For Indigenous Peoples, including First Nations Australians, self-determination is a human right that is necessary in all aspect of their lives. Furthermore there is a paucity of published literature regarding Indigenous Peoples self-determination in alcohol policy development [20].

**Fig. 1:**
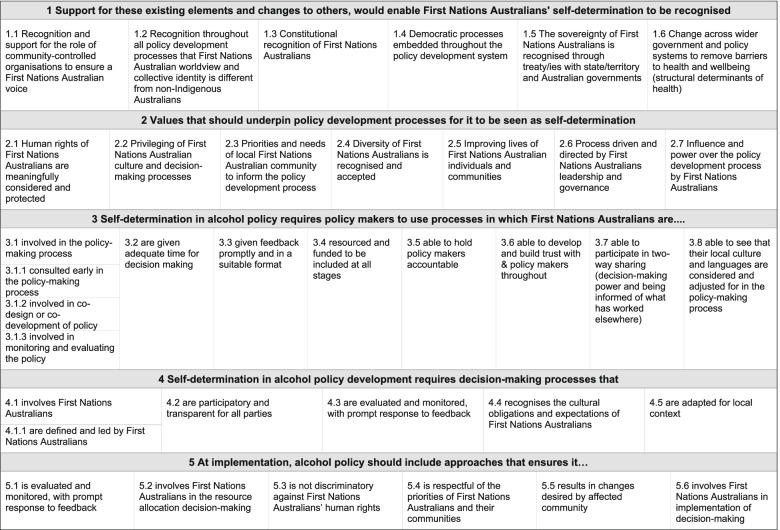
A framework of elements needed for self-determination in the development of alcohol policy (adapted from [25])

To progress the literature, we conducted a Delphi study with First Nations Australian experts to identify the elements needed for First Nations Australians’ self-determination in health and alcohol policy development [25]. The current study applies this framework (Fig. 1) to the second largest town in the Northern Territory, Alice Springs/Mbantua [25]. This study aims to identify the extent to which First Nations Australians perceive they have experienced self-determination in relation to current alcohol policy in Alice Springs/Mbantua in the Central Australian region of the Northern Territory.

## Methods

### First Nations Australian leadership

This study was led by AES, a Nyungar 3 woman; although based in Western Australia (WA) since 2004, she has worked, and intermittently lived in, Alice Springs/Mbantua [26]. AES has conducted a number of evaluations of alcohol and other drug interventions led by First Nations Australians in Central Australia [27, 28, 29, 30]. A priority of this work was to build and support the research capacity of the Central Arrernte peoples, who are the traditional owners for Alice Springs/Mbantua [31].

### Ethical approvals

Ethical approval was provided by Curtin University Human Research Ethics Committee (HRE2019-0729) and the Central Australian Human Research Ethics Committee (CA-19-3525). Participation was opt-in and voluntary. Both verbal and written consent was sought. Participants were offered a gift-card (supermarket gift-card value $40), in appreciation of their time [32].

### Setting

As the historical and cultural context is critical to this topic, some detail will follow. The Northern Territory is Australia’s third largest and least populated mainland state/territory (1.4 million km^2^); comprising just 1% of the national population (229,000) [33]. Alice Springs/Mbantua is the main town for the Central Australian region (549,564 sq km) [34], and second largest outside of the capital city (Darwin). The Central Arrernte peoples refer to Alice Springs as “Mbantua” [31], and this term will be used here. Mbantua is a traditional meeting place and primary service centre for the Central Australian region. More than one-third (36%) of Central Australian residents identify as First Nations Australian and speak one or more of six prominent First Nations Australian languages [31, 33].

In addition to suburban housing, Mbantua has 18 town camps. These camps were originally on the outskirts of Mbantua and where First Nations Australians stayed while visiting the town, but have become multi-generational ‘suburbs’ with permanent housing [31]. As a result, Mbantua’s population is highly transient and likely much greater than indicated by census figures [34]. Since 1978, the NT has been self-governing; however, as a territory (rather than a state) the Australian Government can override or impose any legislation made by the NT Government. For example, the overruling of the *Rights of the Terminally Ill Act, 1995* (NT) [35] (voluntary assisted dying legislation) with the *Euthanasia Laws Act, 1997* (Cth) [36].

### Overview of NT alcohol policy

Several layers of alcohol-related legislation and by-laws are currently active in the NT (Table 1). In 2016, following years of reactionary and punitive alcohol-related legislation (e.g. *Alcohol Mandatory Treatment Act*, *2013* (NT)) [16, 37, 38, 39], the newly-elected Labor government initiated the Riley Review of the NT alcohol policies [40]. Following 138 written submissions^4^ and public consultations in 21 towns and communities, 220 recommendations were made by the review panel (which included one First Nations Australian woman) [40]. 

The NT Government supported (*n* = 186) or gave ‘in-principle support’ (*n* = 33) for the majority of recommendations. The only recommendation not supported was the cessation of Sunday take-away trading [40, 41]. 

As the result of the Riley Review, NT alcohol policy and legislation has been systematically reformed. Two new alcohol-related acts were implemented – the *Alcohol Harm Reduction Act 2017* (NT) [42] and the *Liquor Act 2019* (NT) [43]. Both pieces of legislation apply to the entire NT, including visitors. However, these reforms were also required to comply with the *Stronger Futures in the Northern Territory Act*, *2012* (Cth) [44] (Stronger Futures). Numerous reforms were made, including a minimum unit price for take-away alcohol ($1.30 per standard drink; the first Australian jurisdiction to do so) [45], re-introduction of the amended Banned Drinkers Register [46], and formalising the presence of Police Auxiliary Liquor Inspectors outside retail liquor outlets in three large regional towns – Alice Springs/Mbantua, Tennant Creek/Anyinginyi, and Katherine [39, 41].

#### Stronger Futures in the NT

In 2007, the Australian Government suspended the *Racial Discrimination Act, 1975* (Cth) [47] in the NT to impose the *Northern Territory National Emergency Response Act*, *2007* (Cth) [48] (‘NT Intervention’) [49, 50]. In 2012, the NT Intervention was superseded by the *Stronger Futures Act in the Northern Territory 2012* (Cth) [44]. Both legislations were applicable to residents and visitors of ‘prescribed areas’ in the NT (affecting an estimated 70% of NT First Nations Australians) [50]. Prescribed areas consisted of lands under the *Aboriginal Land Rights (Northern Territory) Act 1976* (Cth) [51], *‘*town camps’ in urban centres, and anywhere else deemed by the Minister for Families, Community Services and Indigenous Affairs. It applied to 600,000 km^2^ of the NT (42%), including 500 First Nations Australian communities [50].

**Table 1:** Alcohol-related legislation in Mbantua (Northern Territory, Australia) active as at March 2022 (adapted from [38])

Relevant Legislation	Date in effect from	Implemented by	Agency responsible	Target group	Key elements
Stronger Futures In The Northern Territory Act 2012	1/07/2012	Australian Government	Prime Minister and Cabinet	First Nations Australians living in prescribed areas	
Stronger Futures In The Northern Territory (Alcohol Management Plans)	1/02/2013	Australian Government	Prime Minister and Cabinet	First Nations Australians living in prescribed areas	• Locally developed plans to manage the harm, demand, and supply of alcohol in addition to this legislation • Sunset date - for legislation to cease
NT Liquor Act Public Restricted Areas Legislation	1/08/2007	Alice Springs Town Council – NT Liquor Commission		Entire Mbantua population	• Mbantua declared a dry town under the NT Liquor Act Public Restricted Areas legislation.
Alcohol Harm Reduction Act 2017	1/09/2017	Northern Territory Government	Department of Health	People making takeaway alcohol purchases	• Banned drinkers order • Income management
Liquor Act 2019	1/10/2019	Northern Territory Government	Department of Industry, Tourism and Trade	People making takeaway alcohol purchases First Nations Australians living in prescribed areas (SF Compliance)	• Systematically rescinding previous legislation • Minimum unit price for take-away ($1.30 per standard drink) • Introduction of a reformed Banned Drinkers Register • Restrictions on liquor licences • Prohibited public places (dry areas) • Point of sale restrictions • Measures to comply with Stronger Futures

Criminalising possession of alcohol in prescribed areas was a key focus of the NT Intervention and Stronger Futures. However, this led to existing alcohol restrictions being overridden in more than 100 First Nations Australian communities across the NT, the majority of which were in Central Australia [37, 52, 53, 54]. Two significant amendments to alcohol policy were enforced under Stronger Futures: [i] harsher penalties for possession of alcohol in prescribed areas (fines of more than $74,000 and/or 18-months in prison), and [ii] community-developed alcohol management plans (AMPs) [40]. AMPs needed to comply with NT and Australian Government legislation and required approval from the Federal Minister for Indigenous Affairs. In 2016 these requirements were amended due to implementation difficulties. D’Abbs [55] described the barriers faced by an NT community in getting an AMP approved including changes in Government, the minister responsible, the requirements and the legislation. By late 2015 just one AMP had been approved [56]. As a result of the Parliamentary review, the Australian and NT Governments partnered to implement community-led ‘Alcohol Action Initiatives’ [37, 40]. The Initiatives are short-term partnership projects with First Nations Australian communities to implement locally led supply, harm, or demand reduction strategies [39, 57].

#### Alcohol policy in Mbantua

In addition to NT-wide measures, locally specific alcohol policy measures also applied in Mbantua. Since 2006, numerous local AMPs have been introduced [58], the most notable of which was the 2007 AMP that declared Mbantua to be a “dry town” under the restricted areas of the *Liquor Act 1978* (NT) [59]. “Dry” areas or towns use provisions within NT legislation to prohibit the possession or consumption of alcohol within a defined area [60, 61]. In 2008, Mbantua was the first NT location to introduce scanning of identification at point-of-sale in liquor outlets [58, 62]. In 2014, starting as Temporary Beat Locations, police officers were stationed outside liquor outlets to ask people purchasing alcohol their place of residence [16, 63]; a measure now embedded in the *Liquor Act 2019* (NT) [43]. Cumulatively these factors bring unique challenges for all Mbantua residents when navigating local liquor regulations [58, 62].

### Participant recruitment

A multi-staged convenience sample was used to recruit First Nations Australian community members and key stakeholders. Eligibility criteria were: able to legally purchase alcohol (18 years or older); living in Central Australia; and, identifying as First Nations Australians. Participants were invited if they were: [i] community leaders who have advocated or supported community-led alcohol measures; [ii] past and current leaders and/or staff of Central Australian-based Aboriginal community-controlled organisations (including health); and [iii] community members with experience of the current alcohol policy.

To initiate the study, AES visited Mbantua in February 2020 to discuss the study scope and purpose with key community members (*n* = 9; seven First Nations Australians), and to identify key individuals who could be involved. Agreement was made with local Arrernte researchers and key staff of a local community-controlled organisation to conduct interviews in April 2020. However, interviews were postponed due to Covid-19 travel restrictions between Western Australia and the Northern Territory [64]. When WA’s Covid-19 restrictions eased in December 2020, AES visited Mbantua (over 3 days) to connect with possible participants, discuss the proposed interview approach, and identify appropriate timing for interviews.

Interviews were conducted in English (by AES) over 16 days in Mbantua (March 2021). Prior to arrival, invitations for interviews were emailed (by AES) to key community members and leadership of Aboriginal community-controlled organisations (*n* = 18), including five individuals with whom AES had existing professional relationships. While in Mbantua, three participants did not respond to follow-up phone calls, and so no further contacts were made. An additional eight participants were recommended by other participants, of whom two agreed to participate, and one brought another three participants with them for a group interview.

### Interviews

#### Yarning

The interviews were conducted using a ‘yarning’ method [65]. Yarning is a conversational approach to interviewing that allows for the authority and foundations of the knowledge and social systems of First Nations Australians, founded on a shared understanding of relationships and accountability between all involved [65, 66]. Bessarab and Ng’andu (2010) describe three key components of yarning in a research context: (i) social yarning (to connect and establish relationship); (ii) research topic yarning (focused on experience of current alcohol policy and self-determination in Mbantua); and (iii) collaborative yarning (where solutions were discussed) [67]. The interview yarns varied depending on the experience and role of interviewees [67]. A semi-structured schedule was developed to help direct the yarn if necessary, however the conversations were participant-led [67].

Interviews were conducted in a variety of locations (e.g. public spaces, places of employment, and individuals’ homes by invitation) and when convenient for each participant (between 9:30am and 8:30pm). Interviews were audio recorded and transcribed by the interviewer [AES] and an Anaiwan (a First Nations Australian nation in the jurisdiction of New South Wales) research assistant. Interviews ranged in duration from 20 to 90 min (average length: 47 min). Transcripts were de-identified and pseudonyms given to all individuals and organisations mentioned.

### Data analysis

Interview transcripts were imported into NVivo 12 [68] and de-identified. The framework of self-determination in a health (and alcohol) policy context, delineated in Fig. 1, was used as the lens for analysis. This framework was developed through a Delphi study with involvement from 20 Australian experts (*n* = 9 First Nations Australians) [25]. Framework elements were operationalised for use in this study (by AES) – into overarching themes (*n* = 5), elements (*n* = 32), and sub-elements (*n* = 4). Each item of the framework was imported into NVivo12 as a node (or theme) and arranged according to a hierarchy. Three additional nodes were added under every item – to code if the interview mentioned the element, and the context of the mention (present, neutral, or absent).

Interviews were coded in three stages: [i] for evidence of an element of self-determination mentioned within current NT alcohol policy; [ii] confirmation the evidence supported the presence or absence of self-determination, or if the element was discussed as being important but not present or absent (neutral); and [iii] coding verification. A sample of de-identified coded statements (25%) were provided to co-author KSKL with 98% agreement. The one code where there was disagreement, this was discussed, and agreement reached. Once coding was verified, number of interviews (not participants) mentioning the element were collated against each element, and the percentage of interviews (not participants) mentioning the element were presented in Table 3.

## Results

### Participants

Twenty-one First Nations Australians aged at least 18 years and living in Central Australia participated in this study (Table 2). More than half of the participants were women (*n* = 12, 57%) and aged over 50 years (*n* = 11, 52%). Almost 40% (*n* = 8/21) of participants had expertise in advocacy of community-led alcohol measures, and one-third have held leadership roles in local First Nations Australian community-controlled organisations (*n* = 7/21). Six in ten participants were known to AES prior to the study (*n* = 13/21). Eleven participants were approached directly, and 10 were referred to the study by another participant. Most interviews were conducted face-to-face (*n* = 18/21; 86%), with the remainder conducted via phone or video conference (*n* = 3). Face-to-face interviews (*n* = 12) were comprised of one-on-one (*n* = 8), and group interviews (*n* = 4) conducted with between two and four participants (total group interview participants: *n* = 10).

**Table 2:** Characteristics of First Nations Australian participants (*n* = 21)

		Number
**Sex**		
Female		12
Male		9
**Age group**		
18–30		3
31–50		7
51+		11
**Type of interview**		
One-on-one		11
Group (2–4 participants) (*n* = 4)		10
**Relevant prior experience** ^**a**^		
Lives in a prescribed area (themselves or close family)^b^		8
Elders/ community leaders		4
Advocacy or support of community-led alcohol measures		5
Leadership in an Aboriginal community-controlled organisation (past or current)		9
Staff member in an Aboriginal community-controlled organisation (current)		13
Experience in delivering health service provision in the NT		9
Government employee (past or current)		3
**Drinking status**		
Current drinker		15
Non-current drinker ^c^		6

^a^ Some participants had a range of prior experiences that were relevant to the study

^b^ Mentioned living, or having close family living within prescribed areas

^c^ May include participants who have never consumed alcohol

#### Elements of self-determination discussed in interviews

Overall, 20 participants (*n* = 14 interviews) shared their experiences of current alcohol policy in Central Australia drawing on all themes from the framework of First Nations Australians’ self-determination in alcohol policy. One participant focused on their professional role in Mbantua and made no mention of the elements of self-determination contained in this framework. Table 3 shows the proportion of interviews that mentioned each element, and the related context within current alcohol policy in Central Australia (as being: present, absent, neutral). Selected quotes from interviews representing the context of each mention (present, absent, neutral) are presented in Table 4.

**Table 3:** Proportion of interviews discussing elements of First Nations Australian self-determination in alcohol policy

			N	Present	Neutral
**1 Support for these existing elements and changes to others, would enable First Nations Australians’ self-determination to be recognised**					
1.1 Recognition and support for the role of Aboriginal community-controlled organisations to ensure a First Nations Australia voice			6	33%	33%
1.2 Recognition throughout all policy development processes that First Nations Australian worldview and collective identity is different from non-Indigenous Australians			3	-	-
1.3 Constitutional recognition of First Nations Australians			1	-	-
1.4 Democratic processes embedded throughout the policy development system			-	-	-
1.5 The sovereignty of First Nations Australians is recognised through treaty/ies with First Nations Australians and state/territory and Australian governments			-	-	-
1.6 Change across the wider government and policy systems to remove the barriers to health and wellbeing (structural determinants of health)			1	-	-
**2 Values underpinning policy development processes for it to be seen as self-determination**					
2.1 Human rights of First Nations Australians are meaningfully considered and protected			7	-	14%
2.2 Privileging of First Nations Australian culture and decision-making processes			4	-	25%
2.3 Priorities and needs of local the First Nations Australian community inform the policy development process			7	14%	43%
2.4 Diversity of First Nations Australians is recognised and accepted			7	-	43%
2.5 Improvement of First Nations Australian individuals’ and communities’ lives			1	-	-
2.6 Process driven and directed by First Nations Australians leadership and governance			3	-	-
2.7 First Nations Australians have influence and power over the process			3	-	-
**3 Self-determination in alcohol policy requires policy makers to use processes in which First Nations Australians are….**					
3.1 involved in the policy-making process			8	-	13%
3.1.1 consulted early in the policy-making process			-	-	-
3.1.2 involved in co-design or co-development of policy			-	-	-
3.1.3 involved in monitoring and evaluating the policy			4	-	-
3.2 are given adequate time for decision making			-	-	-
3.3 given feedback promptly and in a suitable format			6	-	33%
3.4 resourced and funded to be included at all stages			-	-	-
3.5 able to hold policy makers accountable			3	-	-
3.6 & policy makers can develop and build trust throughout			-	-	-
3.7 two-way sharing (decision-making power and being informed of what has worked elsewhere)			-	-	-
3.8 local culture and languages are considered and adjusted for in the policy-making process			5	-	20%
**4 Self-determination in alcohol policy development requires decision-making processes that**					
4.1 involves First Nations Australians			6	17%	-
4.1.1 is defined and led by First Nations Australians			4	-	-
4.2 are participatory and transparent for all parties			4	-	-
4.3 are evaluated and monitored, with prompt response to feedback			4	-	-
4.4 recognises cultural obligations and expectations of First Nations Australians			4	25%	-
4.5 are adapted for local context			-	-	-
**5 At implementation, alcohol policy should include approaches that ensures it…**					
5.1 is evaluated and monitored, with prompt response to feedback			5	-	20%
5.2 involves First Nations Australians in resource allocation decision-making			1	-	-
5.3 is not discriminatory against First Nations Australians’ human rights			12	-	8%
5.4 is respectful of the priorities of First Nations Australians and their communities			11	27%	-
5.5 results in changes desired by the affected community			8	12%	-
5.6 involves First Nations Australians in implementation decision-making			7	14%	-

Some interviews had evidence elements were both present and absent simultaneously

**Table 4:** Selection of quotes evidencing the presence or absence of each element of self-determination framework

Present	Neutral	Absent
**1 Support for these existing elements and changes to others, would enable First Nations Australians’ self-determination to be recognised**		
1.1 Recognition and support for the role of Aboriginal community-controlled organisations to ensure a First Nations Australia voice		
…*community-controlled organisation, it’s not like people just sit back passively … people know they can go to … a board member and have it chat to them.* (Community leader #3, female, aged > 51 years)	*…you just can’t say that having a board of governance is the only pathway of involving the Aboriginal voice. And that’s because the capturing of policy is driven by the white fathers in the institution… that alone is not going to solve our problems … [you need to] create this pathway and a model … To engage Aboriginal peoples voice on matters.* (Community leader #2, female, aged > 51 years)	*… [community-controlled boards] that structure is not a structure …. [it is] there to be able to access government funding … that assimilation model is just the formation of Western model just so that we can access funding … It’s not our structure … Aboriginal people don’t have a hierarchy structure.* (Community leader #1, male, aged > 51 years)
1.2 Recognition throughout all policy development processes that First Nations Australian worldview and collective identity is different from non-Indigenous Australians		
***-***	*-*	*… there is institutionalised racism occurring right across every agency … you’re applying two different approaches or laws within the one system … they’re not respecting the blackfella Community, blackfella culture, way of life.* (Community leader #4, male, aged > 51 years)
1.3 Constitutional recognition of First Nations Australians		
*-*	*-*	… *let the Aboriginal leaders of that nation sought out who their boss is. And if we followed the United Nations structure, we would be more equal.* (Community leader #2, female, aged > 51 years)
1.4 Democratic processes embedded throughout the policy development system		
-	-	-
1.5 The sovereignty of First Nations Australians is recognised through treaty/ies with First Nations Australians and state/territory and Australian governments		
-	-	-
1.6 Change across the wider government and policy systems to remove the barriers to health and wellbeing (structural determinants of health)		
*-*	*-*	… *[There] needs to be systemic changes at a government level … I just think it’s almost impossible … it’s a big ship going through the ocean, you can’t turn it … it’s a whole social, political, social economic thing which is driven by whitefellas, and that’s why it’s not going to change.* (Community leader #4, male, aged > 51 years)
**2 Values underpinning policy development processes for it to be seen as self-determination**		
2.1 Human rights of First Nations Australians are meaningfully considered and protected		
*-*	…*not only black people…[have] drinking problems … white people … just don’t show their problems out in the open.* (Community members #20/#21, females, aged 18–30 years)	*… I feel it’s just like the intervention was, very controlling. This is very controlling, and what’s next? … what are they gonna control next in our lives?* (Community member #11/#12, female/ male, aged 31–50 years)
		*… they [are] saying it’s not racial, but … you can see how racial it is, like, when you go to line up to go in the bottle, or you know, you got to show your ID. You see, white mob just walk straight through.* (Community members #20/#21, females, aged 18–30 years)
2.2 Privileging of First Nations Australian culture and decision-making processes		
*-*	*… we have to realise that just having a structure for input from organisations and leaders that are in the organisation is not the only voice [but we] promote the governance structure as being the voice of the people.* (Community leader #2, female, aged > 51 years)	*… they [Aboriginal people] should be more involved in decision-making because it’s all about them … They know their cultural decision-making and all that stuff … there’s a lot of people around Alice Springs, in the town camps, [that] can speak up.* (Community member #6/#7, females, aged > 51 + years)
2.3 Priorities and needs of local the First Nations Australian community inform the policy development process		
*… we were really frustrated … they needed to prioritise police on the outlets [for the community] … it’s a strategy that has had the sort of biggest impact in terms of reducing alcohol consumption and harm … our board was in support of that.* (Community leader #3, female, aged > 51 years)	*… we’ve got to find a pathway of developing the people [First Nations Australians] and their voice because we’ve left them behind … they’ve become recipients.* (Community leader #2, female, aged > 51 years)	… *[specific organisation] don’t even talk to [their members] so they act in isolation … It’s unaccountable. It’s their own volition … there is no community consultation.* (Community leader #1, male, aged > 51 years)
2.4 Diversity of First Nations Australians is recognised and accepted		
*-*	*… that’s hard, because you’ve got you know, Alice Springs is not just our, like, residential. We’ve got our town camps, which are prescribed areas you can’t consume alcohol there.* (Community member #10, female, aged 31–50 years)	*… And they are forgetting this could be their third language English.* (Community members #14–#17, group males, aged 18 + years)
		*Those people [policy makers] know nothing about … The people they prescribe policy for … What would those people know about my life as a stolen generation [person]? … They have food in the fridge, they have a roof over their head, they have a warm bed every night. They have a car to go to work, they have money in the bank, they are able to pay their bills … these are people with power and privilege who talk about people they know nothing about.* (Community leader #1, male, aged > 51 years)
2.5 Improvement of First Nations Australian individuals’ and communities’ lives		
*-*	*-*	…*[there’s] some consequences for people that live in urban housing … if you say no to a family member that you can’t buy alcohol … that person will no longer classes you as family and pushes you aside and that really hurts … or they end up smashing the house or stealing stuff.* (Community member #6/#7, females, aged > 51 + years)
2.6 Process driven and directed by First Nations Australians leadership and governance		
*-*	*-*	… *we’ve left them [First Nations Australians] behind … they’ve become recipients and data is a way of driving policy change … I think it’s healthy, to find facilitate ways of having input.* (Community leader #2, female, aged > 51 years)
2.7 First Nations Australians have influence and power over the process		
*-*	*-*	*I think making decisions for Aboriginal First Nations Peoples needs to include us – First nations people. If it’s got to be dealing with Aboriginal Peoples issues, it’s got to come from Aboriginal People. It’s not for the non-Indigenous people to making decisions for [us].* (Traditional owner #8, female aged > 51 years)
**3 Self-determination in alcohol policy requires policy makers to use processes in which First Nations Australians are….**		
3.1 involved in the policy-making process		
-	*to have a voice heard … [but] Which is voice to be heard … It’s complex … not one institution is really going to be structurally able to address this situation means people have to come together.* (Community leader #2, female, aged > 51 years)	*… Aboriginal men have been excluded from being part of the process mainly because I don’t want to talk to white people about drinking … men are powerless [in] the way policy is developed.* (Community members #14–#17, group males, aged 18 + years)
		*[The] government sort of dictating to us and overruling the rights for us to either purchase alcohol or drinking in an environment where you are allowed to.* (Community member #6/#7, females, aged > 51 + years)
3.1.1 consulted early in the policy-making process		
-	-	-
3.1.2 involved in co-design or co-development of policy		
-	-	-
3.1.3 involved in monitoring and evaluating the policy		
-	*-*	… *I’d like to know how long this BDR [banned drinkers register] thing gonna go [on for]. And I would like to know if they need feedback to community people on the findings of the data, and what are they going to use it for? And are they continue with all this stuff, putting pressure on people living in urban housing.* (Community member #6/#7, females, aged > 51 + years)
		*Did you know how many non-Indigenous IDs [identification] has been tracked, compared to an Indigenous ID?* (Community member #6/#7, females, aged > 51 + years)
3.2 are given adequate time for decision making		
-	-	-
3.3 given feedback promptly and in a suitable format		
	*It’s not just Aboriginal people. It’s across the board … if you were to break it down stat by stat … the facts are this percentage of people are committing alcohol-related offences.* (Community member #13, male, aged 31–50 years)	*Female 1: Did anybody get any feedback from the data that’s been collected?…* *Female 2: According to the data … it’s smooth. It’s all down … And it’s all under control. Not all the policy that they put in place for us does work. Because [they don’t] know what it’s like to live in a town camp.* (Community member #6/#7, females, aged > 51 + years)
3.4 resourced and funded to be included at all stages		
-	-	-
3.5 able to hold policy makers accountable		
-	-	…*Aboriginal people aren’t allowed to say how they want to address it … we shouldn’t be measuring the impact of alcohol policy on Aboriginal people by looking at … [it] needs to be measured [in the way that Aboriginal] people want to have the policy or how it’s going to be useful to them.* (Community member #5, female, aged > 51 + years)
3.6 & policy makers can develop and build trust throughout		
-	-	-
3.7 two-way sharing (decision-making power and being informed of what has worked elsewhere)		
-	-	-
3.8 local culture and languages are considered and adjusted for in the policy-making process		
*-*	…*[community-control] becomes captured by corporate governance, which is whitefella law … Aboriginal law and leadership operates in a totally different construct … within that Aboriginal construct, there are layers and different voices.* (Community leader #2, female, aged > 51 years)	*Female 1: Like you can’t even go sit down in town camps anymore with your family because they’ve got the restrictions there.* *Female 2: Yeah, all the time. You could be sitting at a big bunch outside just talking story and then [police pull up], question everything in the gammin and search around.* (Community members #20/#21, females, aged 18–30 years)
**4 Self-determination in alcohol policy development requires decision-making processes that…**		
4.1 involves First Nations Australians		
… *[we’ve had] dedicated workshops on alcohol policy … what are our views? what is everyone’s views about a floor price? … what are the things that are good and need changing?* (Community leader #3, female, aged > 51 years)	*-*	*They don’t talk to people … It’s not hard to sit down and talk to people, because people we all got the same problems … we’ve all got to work with people to make this town a better place for people live in.* (Traditional owner #8, female aged > 51 years)
		*… there was a level of Aboriginal input, I wouldn’t call it self-determination because there never was self-determination.* (Community leader #4, male, aged > 51 years)
4.1.1 are defined and led by First Nations Australians		
*-*	*-*	*We [First Nations Australians] should be all pulling together making decisions … it’s not about people, non-indigenous people mistrusting First Nations people …. it’s like the laws are all packed up against [us].* (Traditional owner #8, female aged > 51 years)
4.2 are participatory and transparent for all parties		
-	-	*Female 1: … it’s all to do with what the what non-Indigenous mob bring in without consulting the Indigenous mob … then when Indigenous people trying to make the changes and present something to you know, we don’t know if it gets heard or if it’s not even relevant for them little agenda …* *Female 2: … there are a lot of non-Indigenous, as well as some Indigenous, that say … this doesn’t work … You never hear government say “oh, that didn’t work” …* (Community member #6/#7, females, aged > 51 + years)
4.3 are evaluated and monitored, with prompt response to feedback		
*-*	*-*	*… we all switch off because we’re sick and tired of hearing [from] that person.* (Community members #14–#17, group males, aged 18 + years)
		*Female 1: So is there is there is there anything to say that this thing is working? You know, you say more or less alcohol is sold* *Female 2: no feedback* *Female 1: …how much alcohol is sold every month.* (Community member #6/#7, females, aged > 51 + years)
4.4 recognises cultural obligations and expectations of First Nations Australians		
…*The upside to it is that people know, they can go to … a board member … That’s what I think is really fantastic about community-controlled organisations.* (Community leader #3, female, aged > 51 years)	-	*… [First Nations Australians] need to know that they can have autonomy. And you can still speak … As a leader in your own right … some of these institutions do have good connections to people in the community and not everybody wants to speak about issues.* (Community leader #2, female, aged > 51 years)
		*Are you going to be sharing it with anyone? … when I’m drinking my family would drink me … I rather [they are] drinking at my place and then crashin’ here.* (Community member #19, female. Aged 18–30 years)
4.5 are adapted for local context		
-	-	-
**5 At implementation, alcohol policy should include approaches that ensures it…**		
5.1 is evaluated and monitored, with prompt response to feedback		
*-*	*Male: … they said … statistics [have] come down.* *Female: [But] you wouldn’t say it’s significantly… impacted and made a huge difference.* (Community member #11/#12, female/ male, aged 31–50 years)	*And there’s a responsibility of the Indigenous organisations to ensure that their constituents know precisely what it is. From in town … and out bush, which means it’s got to be done in language.* (Community leader #4, male, aged > 51 years)
5.2 involves First Nations Australians in resource allocation decision-making		
-	-	*… they [have] basically taken away responsibility in any policy development initiatives, including alcohol, taken away [those controls] at a local level … It’s got to be run by Indigenous people delivered by Indigenous people and based on Aboriginal cultural considerations.* (Community leader #4, male, aged > 51 years)
5.3 is not discriminatory against First Nations Australians’ human rights		
*-*	… *I believe that the rules of not allowing people to purchase alcohol [are appropriate] … Every single person on Centrelink should not be allowed to purchase alcohol … if you want to drink, you work like the rest of us, and then you’ve earnt it.* (Community member #10, female, aged 31–50 years)	*… we just need to be informed and empowered of certain information … You know, human rights.* (Community member #19, female. Aged 18–30 years)
		… *[it doesn’t] matter you’re working 37.5 (hours), you’re paying a mortgage, or you live in a public housing … If you’re Aboriginal, you’re in that group … Aboriginal people are targeted and that’s unfair.* (Community member #6/#7, females, aged > 51 + years)
5.4 is respectful of the priorities of First Nations Australians and their communities		
…. *I want it to be a bit more strict. I reckon if you get banned from for take-away, you should be banned from going into the pub.* (Community members #14–#17, group males, aged 18 + years)	*-*	*… it’s okay to say let’s turn the tap down, but what about the trauma that’s being left for communities to try and deal with people that have an addiction … we can’t just turn the tap down without ignoring the underlying cause … it is a symptom.* (Community leader #2, female, aged > 51 years)
5.5 results in changes desired by the affected community		
… *I guess it’s [the Banned drinkers register] a double-edged sword. you asked women … and they will tell you that the BDR did what it was meant to do … It keeps him [husband] from spending the money on the grog.* (Community member #10, female, aged 31–50 years)	*-*	*Female 1: … this will bring in more pressure on house owners, people that live in a public [housing]* *Female 2: … and it’s actually dividing families.* *Female 1 … So who’s responsible for that consequence?… house-owner or house-boss of that urban house could end up going crazy because of all that pressure from family members and cause the violence this is what nobody is not thinking about. The consequences of what happened … the pressure they put on their family members.* *(Community member #6/#7, females, aged > 51 + years)*
5.6 involves First Nations Australians in implementation decision-making		
*… [with] # members; we’ve got a board that’s elected or appointed by the members. So, any policy on alcohol has to go to the board … basically [that] sets the parameters around Aboriginal engagement for the organisation around alcohol policy.* (Community leader #3, female, aged > 51 years)	-	*… we don’t want white people speaking on our behalf anymore. It’s not white people’s issue, they can hide it. But or mob, all of us get humbugged every day.* (Community members #14–#17, group males, aged 18 + years)

As shown in Table 3, 12 of the 36 elements (including 2/4 sub-elements) were not mentioned in the interviews at all. Of the elements that were mentioned, just 20% (*n* = 7/36) were ‘present’. In contrast, 75% of the elements were absent from current alcohol policy processes (*n* = 27/36). Just over a quarter (28%) of elements were seen to be important, but as no mention was made as to it being present or absent, these were coded as ‘neutral’ (*n* = 10/36).

##### Support of systemic elements needed for recognition of First Nations Australians’ self-determination

At the top level of this framework, there are six systemic (macro-level) elements needed for self-determination to occur. Five participants (all with experience in leading Aboriginal Community Controlled Organisations or ACCOs 5) made 16 mentions of four of the six elements. The two elements that were not mentioned were – the need for democratic process to be embedded in the policy development system (1.4) and the need for First Nations Australians’ recognition of sovereignty through treaties (1.5). The recognition of First Nations Australian worldview (1.2), constitutional recognition (1.3), and addressing of structural determinants of health (1.6) were all mentioned by participants as needed but absent from current policy processes. Participants were mixed in relation to the importance and support of ACCOs (1.1). Interviews mentioning this element were evenly distributed and discussed the role and importance of ACCOs in providing a First Nations Australian voice to policy processes.

##### Values underpinning policy development processes needed for self-determination

Of the seven key values (elements) necessary for self-determination, 13 interviews (*n* = 19/21 participants) mentioned at least one element. Overall, these elements were mostly described as being absent from current alcohol processes. The only element present (*n* = 1/10) was also the one with the most mentions of it being needed (Neutral; *n* = 5/10; 2.3; priorities of local community to inform the process). Three elements were mentioned as absent or needed – consideration of First Nations Australians’ human rights (2.1), importance of culture (2.2) and recognition of diversity (2.4). The remaining three elements were mentioned as being absent for First Nations Australians – lives’ to be improved (2.5), to direct the process (2.6), and to have influence over policy (2.7).

##### Elements needed for the alcohol-related policy development process

The next level of the framework presents elements necessary for the overall policy development process. Twelve interviews (18 participants) mentioned eight elements and three sub-elements. Overall, this theme had the greatest proportion of elements which were not mentioned in the interviews (*n* = 6/11; 67%). Two elements were mentioned as absent from the current policy processes – First Nations Australians involvement in evaluation of policy (3.1.3) and being able to hold policymakers accountable (3.5). The remaining three elements were mentioned in the context of being both absent and needed (neutral) – the policy-making process should: involve First Nations Australians (3.1), have a feedback process (3.3) and be adjusted for the local culture (3.8). Within this theme, 85% of mentions discussed First Nations Australians as being absent from the policy development process (*n* = 29/34).

##### Decision-making elements for self-determination in alcohol policy development

The next level of the framework presents elements that focus on decision-making in alcohol policy development processes. Twelve interviews (*n* = 18 participants) mentioned five of these elements and one sub-element. The only element not mentioned was decision-making that has been adapted for local context (4.5). Two elements were both present and absent – decision-making involvement (4.1) and recognition of cultural obligations of First Nations Australians (4.4). Three elements were absent from the policy development process: decision-making processes led by First Nations Australians (4.1.1), participation from all parties (4.2) and involvement in evaluation with feedback (4.3).

##### Elements needed to implement alcohol policy

Twelve interviews (18 participants) mentioned evidence related to all six elements that were needed for implementation of alcohol policy. Just one element was absent from the current alcohol policy process – First Nations Australians’ involvement in resource allocation (5.2). Two elements were absent and needed (neutral) – implementation should be evaluated (5.1) and not discriminatory (5.3). The remaining three elements were mentioned as being both present and absent in the current context – implementation is: respectful of community priorities (5.4), results in change desired by communities (5.5), and involves First Nations Australians (5.6).

## Discussion

This study qualitatively assessed the degree of self-determination experienced by First Nations Australians in alcohol policy against a framework of elements [25]. This unique framework, was derived from expert opinion in a previous study by this research team, as a broader program of work [25]. The framework was applied to participants’ yarns about their experiences of current alcohol policy in Central Australia. Critically, little evidence was found of self-determination in the participants’ experiences of current alcohol policy. A diversity of experience of self-determination was described, with 19% of elements noted as being both present and absent (*n* = 7/36). Implementation (Theme 5) was the most frequently referenced theme from the self-determination framework. The absence of First Nations Australian leadership and representation were notable.

### Implementation of policy

Implementation was primarily discussed in the context of elements being absent from the current alcohol policy process. Participants spoke of not being consulted or having the opportunity to contribute to the development of current alcohol policies. While the recent Riley Review worked to ensure that First Nations Australians had greater opportunities to contribute to NT alcohol policy than previous policies, there is little detail of the degree to which First Nations Territorians participated in the process [40]. This likely also speaks to the uniquely layered and tangled alcohol policy context for First Nations Territorians [39, 55]. Unlike their non-Indigenous counterparts, First Nations Territorians are required to comply with the *Stronger Futures in the Northern Territory Act 2012* (Cth) [44], in addition to the NT-wide *Alcohol Harm Reduction Act 2017* (NT) [43] and *Liquor Act 2019* (NT) [43]. On face-value the alcohol restrictions in prescribed areas introduced by the Australian Government were similar to community-led ‘dry’ area rules. However, in reality, the NT Intervention replaced the carefully negotiated and locally-constructed alcohol policy measures with blanket punitive penalties [69].

### First Nations Australian leadership

Fundamental to addressing alcohol-related harms is the need for First Nations Australian leadership in alcohol-related policy. However, with alcohol, this is rarely prioritised to the same degree as has been observed for other health issues [37, 70]. A consequence of this lack of leadership is that while current policies may be evidence-based, they do not recognise the specific cultural diversity and uniqueness of the NT population, nor how alcohol-related policies could be facilitating experiences of disempowerment, social exclusion, and racism which in turn have been found to have negative effects on health, including alcohol-related harm [71].

The framework applied in these data (Fig. 1) has a number of elements related to First Nations Australians being involved in or leading the development and implementation of policy (*n* = 12) [25]. The study participants indicated that community-based leadership in Central Australia was absent from current alcohol policy processes. Previous studies have demonstrated the importance of First Nations Australian community leadership in leading policy responses to address alcohol-related harms [15, 72, 73]. For example, First Nations Australian women in Fitzroy Crossing (Western Australia) led efforts to reduce widespread alcohol-related harms [72, 74]. The collaborative process undertaken by these women enabled everyone to contribute to the process [72, 74]. As another example, over nearly a decade (1988–1997), the Ngaanyatjarra Pitjantjatjara Yankunytjatjara (NPY) Women’s Council successfully advocated to reduce supply of alcohol in Curtin Springs (Northern Territory) because of significant alcohol-related harms [75]. In comparison with the Fitzroy Crossing and NPY Women’s Council examples, the absence of any First Nations Australian consultation, let alone leadership, in the NT Intervention and Stronger Futures legislation cannot be ignored [76, 77, 78].

Later amendments to Stronger Futures allowed for First Nations Australian communities in prescribed areas to develop their own AMPs [37, 39]. However, as discussed earlier in this paper, communities that did develop AMPs faced significant impediments, with just one AMP approved by late 2015 [39, 55, 56]. In 2016, AMPs were replaced with Alcohol Action Initiatives, a collaborative partnership between the Australian and NT governments and communities [39, 55, 79]. While current NT Government alcohol-related legislation allows for location-specific measures, such the dry-area rules under the Alice Springs (Mbantua) Alcohol Management Plan [58], communities in prescribed areas must also comply with the Stronger Futures legislation. Overall, this complex landscape does not allow for much space for First Nations Australians to have any leadership in alcohol-related policy.

### Representation

Inclusion of First Nations Australians in the development and implementation of policy also warrants consideration of representation. All the participants who had held leadership positions within First Nations Australian community-controlled organisations (ACCOs), discussed the role of ACCOs as a representative voice. Some participants were supportive and others, while supportive, suggested that ACCOs should not be solely relied on to provide the First Nations Australian perspective. ACCOs grew from a history of communities taking leadership to ensure access to culturally secure and safe care [80]. Recently ACCOs, and their peak bodies have become the pathway for providing a “representative voice” especially in the health-sector [81]. However, the participants in this study highlighted the need to recognise the diversity of First Nations Australian perspectives and the multiple pathways taken to include an entire community [82]. Similarly, Hunt [83] and Thorpe et al., [84] describe effective engagement needed for First Nations Australians to actively participate in the policy development process, from defining the problem to evaluation of outcomes. Dreise and colleagues [82] discussed and explored the nature of First Nations Australians representation in policy, and the related consideration of how representative decision-making occurs in the layered policy development process. This supports findings from our previous studies [20, 25] which found that First Nations Australians’ self-determination, requires representation from the entire community and not just one group. The importance of policy development on a foundation of human rights, which includes self-determination, and cannot be understated [82, 84].

### Implications

The framework used in this study could help assess evidence of First Nations Australians’ self-determination in alcohol and other areas of policy development. However, involvement by First Nations Australians would be required to refine and adapt this framework to suit each context. This could enable communities to take a lead role in monitoring the degree of self-determination present in local policy development processes, rather than it being defined by policymakers. While this study demonstrated an overall absence of self-determination from the context of current alcohol policy in Mbantua (Alice Springs), it does provide some evidence of areas that could be improved for greater engagement of the local First Nations Australian community (e.g., communication of outcomes and progress of current legislation). For policymakers, change is needed throughout the policy development stages – not just when implementing policy – for First Nations Australians’ right to self-determination to be recognised. While these results have identified the absence of self-determination within this context, there is also a need to explore the way that First Nations Australians’ self-determination could be recognised and part of the alcohol policy development process for First Nations Australians in Mbantua.

### Limitations

This study has several limitations. Firstly, the interviews focused on participants’ experiences of current alcohol policy in the NT, however, the framework itself [25] was finalised after the interviews were conducted, meaning that its elements were applied to the interview yarning data retrospectively. As such, participants were not probed about specific elements of self-determination contained in this framework. Although most elements contained in this framework were mentioned in the interviews (*n* = 24/36), the majority of the discussion was on the absence (*n* = 24/36), rather than presence (*n* = 7/36), of framework elements. Despite this, the framework provided a useful independent comparator to gauge the degree of self-determination evident in current alcohol policy in Central Australia. Secondly, a relatively small sample was recruited (*n* = 21) and yarning interviews focused on the experiences of only First Nations Australian community members, not of policy makers or non-First Nations Australian community members. The participants, however, shared their vast experience and knowledge in this study (Table 2). Thirdly, AES’ existing professional relationship with many participants (*n* = 13/21) was a strength and a limitation. While First Nations Australian participants were willing to take part in an interview, the longstanding relationship between AES and participants could be a potential source of bias. All efforts were taken to minimise bias (e.g., the yarning method used in the interview schedule enabled participants to discuss their priorities in relation to current alcohol policy). The existing relationships also ensured that there was both cultural accountability to the local community, and a longstanding relationship founded on reciprocity [85, 86]. Finally, this study was conducted at the height of the Covid-19 pandemic. It is unclear to what extent this had any influence on perceptions of self-determination.

## Conclusion

Alcohol policy for First Nations Australians in the NT, is nuanced and complicated. The self-determination framework used to assess local current alcohol policy processes, while identifying some evidence of First Nations Australians’ self-determination, there were more elements absent. The importance of self-determination and how it contributes to the health and wellbeing of First Nations Australians needs consideration when developing policy. Self-determination is not something that can be simply applied. A conscious approach is needed to recognise and implement the right to self-determination, which must be led and defined by First Nations Australians. To achieve this, in relation to alcohol policy, a shift is needed in the way First Nations Australians and their health needs are considered and recognised.

## Data Availability

The data generated and analyzed during the current study are not publicly available due to small participant numbers, protection of confidentiality, and in line with the ethical approvals from Curtin University and the Central Australian Human Research Ethics Committees.

## Abbreviations

ACCOs: Aboriginal Community Controlled Organisations or ACCOs
AES: Annalee Elizabeth Stearne (Author)
AMP: Alcohol management plans
AUD: Australian dollar
BDR: Banned drinkers register
ID: Identification
KSKL: KS Kylie Lee (Co-Author)
NHMRC: National health and medical research council
NT: Northern Territory
WA: Western Australia

## Footnotes

1 First Nations Australians has been used to collectively refer to the Aboriginal and/or Torres Strait Islander peoples in Australia. First Nations Territorians has been used to refer to those First Nations Australians residing in the Northern Territory (Australia). Where possible to the local language group has been used.

2 Indigenous Peoples has been used in reference to Indigenous Peoples in an international context, specifically those in Australia, Canada, and New Zealand/Aotearoa.

3 Nyungar people are First Nations Australians traditionally residing in the South-west region of Australia (Geraldton to Esperance, Western Australia) [26]. Co-author MW is also a member of the Nyungar nation. MW has a background in social work, and research focuses on culturally secure systems change framework for service providers in partnership with Nyungar elders. Other authors (KSKL, AS, SA) have heritage from outside Australian.

4 Submissions came from many sources including researchers and research institutes, government and Aboriginal community-controlled service providers, and alcohol retailers and suppliers [40].

5 ACCOs: Aboriginal (First Nations Australian) Community-Controlled Organisations are First Nations Australian community-developed and controlled service providers with First Nations Australian community elected boards of governance [87].
